# Dynamic graph structure evolution for node classification with missing attributes

**DOI:** 10.1038/s41598-025-09840-z

**Published:** 2025-07-16

**Authors:** Xiaomeng Song, Bin Zhou, Yanjiang Wang, Weifeng Liu

**Affiliations:** 1 School of Computer Science and Technology, Shandong University of Technology, Zibo, 255000 People’s Republic of China; 2 College of Control Science and Engineering, China University of Petroleum (East China), Qingdao, 266580 People’s Republic of China

**Keywords:** Graph neural networks, Attribute missing, Node classification, Semi-supervised learning, Information technology, Computer science

## Abstract

Graph neural networks (GNN) have achieved remarkable success in various domains, yet incomplete node attribute data can significantly impair their performance. Graph completion learning (GCL) methods have been developed to address this issue, aiming to reconstruct missing node attributes based on existing structural relationships. However, the accuracy of these reconstructions is highly dependent on the quality of the initial graph structure, which often contains errors and inaccuracies. This paper proposes the evolving graph structure (EGS) framework for semi-supervised node classification with missing attributes. EGS dynamically reconstructs the attributes of the nodes and updates the graph structure through an alternating optimization approach. Specifically, we introduce a Dirichlet Energy function with dual constraints to formulate the objective function, which jointly optimizes node structure relationships and attribute reconstruction. Extensive experiments on five benchmark datasets, with different missing rates, and with seven GNN variants demonstrate the effectiveness of EGS, achieving state-of-the-art performance compared to existing GCL methods.

## Introduction

Graph-structured data, which has the flexible representation capabilities of nodes and edges, can effectively model complex systems and be widely used in fields such as literature classification and commodity recommendation^1^. Current mainstream analytical methods for graph-structured data can be broadly divided into two categories: graph fusion methods^2^ and GNN. Traditional graph fusion methods rely on manually designed features. In contrast, GNN achieves automated modeling of graph relationships through end-to-end deep learning frameworks. A core advantage of GNN is their ability to aggregate information from the direct neighbors of any node^3^. This process constructs representations that are rich in features and unique. These representations effectively capture the inherent complex dynamics and interdependencies within graph structures. Additionally, GNN has significant advantages in architectural design. They can simultaneously consider the features of individual nodes and the contextual information provided by their neighboring nodes^4^. This dual focus enables GNN to develop a comprehensive perspective of the graph. The generated representations reflect both the essential characteristics of local node features and the broader global structure of the network.

The effectiveness of GNN is contingent upon a key assumption: the comprehensiveness and accuracy of the node features and structural relationships within the dataset. However, in practical application scenarios, this presupposition is often found to be inadequate. For example, in e-commerce, consumer privacy preferences can obscure critical insights into purchasing behaviors; in citation networks, nonstandardized attributes of academic articles hinder effective attribute extraction; in environmental monitoring, sensor failures can lead to loss of critical data^5,6^. With the emergence of data incompleteness issues, there is a significant challenge to the performance of GNN.

To address this challenge, the field of GCL has sprung up. GCL has achieved significant advances in managing semi-supervised node classification tasks with missing attributes, focusing on reconstructing the absent node attributes based on the alignment between existing structural relationships and the missing attributes. The existing attribute completion methods are primarily divided into two categories: imputation-based attribute completion and model-learning-based attribute completion.

Imputation-based attribute completion methods use reasoning or estimation to fill in missing node attributes. They often use information already present in the graph structure. For example, the neighborhood averaging method^7^ calculates the average of neighbor attributes to complete the data. This process is simple but ignores structural differences. The k-nearest neighbors imputation method^8^ better uses local structural similarities in the graph. However, it is limited by the preset k value and cannot adapt well to graphs with different density distributions. These methods are efficient and easy to implement. However, their linear assumptions and shallow reasoning mechanisms struggle to capture complex nonlinear relationships in graph data.

Model-learning-based attribute completion methods have been developed to address these limitations. They use deep learning models to identify complex relationships between graph structures and attributes, enabling the completion of missing attributes. In recent research, the revisiting initializing then refining method^9^ has improved the accuracy and robustness of attribute completion through high-order structural matrix approximation and initialization optimization strategies. However, initialization still relies heavily on quality. The partial graph convolutional network method^10^ reduces the over-reliance on initialization by using partial aggregation functions and minimizing the model to handle incomplete graph data effectively. However, the framework’s persistent limitation lies in its underutilization of topological relationships inherent in graph-structured data.

The superiority of GCL methods essentially depends on the accuracy of structural relationships within the data. In practical applications, data incompleteness and potential errors in structural connectivity are common. The collected structural relationships often contain incorrect connection information. Such misleading structural relationships can harm the accuracy of completed node attributes and further degrade the performance of downstream semi-supervised node classification tasks. Recently, researchers have started to address this issue. They designed a multi-view graph imputation network^11^, which treats attributes and structures as two views. The contrastive alignment mechanism boosts cross-view consistency in the latent space, yielding enhanced generalizability. However, it decouples attributes from the graph structure, overlooking the issue that substantial attribute changes may lead to structural variations in the graph. Furthermore, compared to methods such as graph convolutional network (GCN)^12^ and other GNN, graph autoencoders are unsupervised learning models. They may not fully utilize existing labels, potentially failing to capture complex features and patterns in graph structures. This can negatively impact downstream task performance.

To address the above challenges, we propose the enhanced graph structure (EGS) method. It targets semi-supervised node classification tasks with missing attributes. The main idea is to dynamically reconstruct node features and update graph structures during the attribute completion process. The core idea is to combine prior knowledge with data-driven learning through multi-regularization joint optimization. This improves the robustness and interpretability of graph completion tasks. Inspired by feature propagation (FP)^13^, we note the advantages of smoothness constraints from Dirichlet energy. However, we also observe that the Dirichlet energy may overly rely on the accuracy of the initial graph structure and potentially ignore the fidelity of attribute data. To address these dual limitations, we develop a unified constraint mechanism that simultaneously optimizes node structural relationship preservation and attribute reconstruction fidelity. This forms a new objective function. In the optimization process of the objective function, traditional methods fix the Laplacian matrix and only optimize the feature matrix. This prevents dynamic correction of the graph structure based on data, limiting the model’s ability to express complex relationships. To address this, we introduce an alternate optimization algorithm. During the correction process, it can dynamically and alternately correct attributes and graph structures. This reconstructs more accurate graph data. The reconstructed node attributes can be applied to downstream semi-supervised classification tasks, significantly improving the performance of GNN in incomplete data scenarios.

The main contributions of this work are as follows:

- We propose a novel evolving graph structure framework for semi-supervised node classification tasks with missing attributes. It highlights the importance of graph structure updates in attribute completion;
- We combine Dirichlet energy with dual constraints for graph structure updates and attribute reconstruction. This provides an effective objective function. During optimization, we introduce a joint linear optimization scheme. It dynamically reconstructs features and updates graph structures through alternating optimization, better completing the graph data;
- Extensive experiments on five widely used benchmarks, five different attribute missing rates and seven GNN variants demonstrate the scalability, and superiority of the proposed method in comparison to the existing GCL methods.

## Related work

### Graph neural networks

GNN is a type of deep learning model designed for graph-structured data. It generates node or graph embeddings by iteratively aggregating information from neighboring nodes. Based on the information aggregation mechanism, GNN are mainly divided into two categories: frequency-domain GNN and spatial-domain GNN.

Frequency-domain GNN defines graph convolution from a signal-processing perspective. It maps graph data to the frequency domain using the Fourier transform, performs filtering operations, and then transforms it back to the spatial domain for feature extraction. Traditional frequency-domain GNN^14–16^ improve performance through convolution and kernel changes, but they require high computational complexity. Recently, the adaptive spectral wavelet transform-based self-supervised GNN^17^ was proposed. It embeds nodes using similar network neighborhoods and features, reducing complexity. However, these methods rely on graph Laplacian matrix decomposition or polynomial approximation, making it difficult to scale to large-scale dynamic graphs.

Spatial-domain GNN directly defines convolution operations in the node domain. It generates the next layer’s node features by aggregating neighbor node features, avoiding frequency-domain transformation. This makes it more suitable for large-scale graph data. GCN is a classic method of spatial-domain GNN. It obtains graph structure information by performing convolution operations on the adjacency matrix, laying the foundation for subsequent research. Graph sample and aggregate (GraphSAGE)^18^ and graph isomorphism network^19^ enhance aggregation, improving performance. However, these methods have high complexity. Recently, a multimodal adaptive spatio-temporal GNN model^20^ multi-dimensional and combines spatio-temporal features in complex spatial data. It improves performance while effectively reducing computational complexity. However, the completeness and accuracy of node features and structural relationships remain key factors affecting GNN performance. In practical applications, missing data is common for various reasons. When data is missing, the performance of GNN can fluctuate significantly.

### Graph completion learning

In the field of graph data mining, one of the core challenges in GCL is the issue of missing attributes. Traditional solutions are mainly divided into two categories: interpolation-based completion and model-learning-based completion.

Interpolation-based completion uses a graph Laplacian matrix-guided k-nearest neighbors imputation method or graph Fourier interpolation for function reconstruction. While existing approaches demonstrate computational efficiency and implementation simplicity, they fundamentally neglect the inherent structural semantics required for comprehensive graph representation learning. Model-learning-based completion employs deep models like graph autoencoder^21^ or GCN for feature reconstruction, effectively alleviating this issue. For example, learning on attribute-missing graphs^22^ combines Transformer and GNN, where the former reconstructs missing features and the latter handles downstream tasks. However, it has high computational complexity and is difficult to extend to heterogeneous graphs. To address this, researchers proposed a higher-order heterogeneous GNN method^23^. It uses a self-attention mechanism transformer to fill missing attributes and adopts an attribute enhancement strategy, enabling the model to fully learn the attributes of heterogeneous neighbors. However, these methods often handle missing attributes separately, neglecting the importance of graph structure learning.

Graph structure learning can effectively constrain feature propagation paths through precise topological connections, improving completion accuracy to some extent. Current graph structure learning methods fall into two typical categories: supervised graph structure learning and unsupervised graph structure learning. Supervised graph structure learning uses known node labels or other forms of supervision to guide the optimization of graph structure. For instance, probabilistic semi-supervised learning via sparse graph structure learning^24^ constructs a semi-supervised graph structure through sparse weighting, effectively integrating unlabeled data. However, node degree issues may affect representation fairness. To solve this, the structural rebalance GNN model^25^ combines graph structure balancing with adversarial learning to improve representation fairness. However, it relies on local labels, which can lead to data distribution skew and edge connection bias. Unsupervised methods alleviate reliance on local labels by using contrastive learning or random walks to mine potential connection patterns. Traditional unsupervised graph structure learning methods^26,27^ are relatively simple but underutilize node features. To address this, the self-constrained graph contrast enhancement network^28^ designs a multi-branch strategy to generate graph structures and combines a feature contrast enhancement module to extract discriminative features. However, this network ignores the fact that updated attribute information can, in turn, promote the optimization of the adjacency matrix. Theoretical analysis reveals that graph topology refinement and node feature recovery inherently exhibit mutually enhancing dynamics through co-evolutionary optimization mechanisms.

Recent research has begun exploring the combination of attribute completion and graph structure learning. For example, the attribute-structure decoupled variational autoencoder^29^ adopts a different strategy: it encodes attributes and structures separately, aiming to learn a shared latent space by maximizing the joint probability distribution between different view representations. However, graph autoencoders may not fully utilize existing labels, making it difficult to comprehensively capture complex features and patterns in graph structures. Additionally, most current methods tend to use static fusion mechanisms, failing to deeply consider other potential effects caused by attribute updates or graph structure adjustments.

### Dirichlet energy

Research related to Dirichlet energy in the field of graph data mainly focuses on GNN optimization and graph structure representation learning. Its core idea is to regulate the smoothness and separability of node features through energy constraints, thereby enhancing model performance. In some graph-related tasks^30,31^, Dirichlet energy has been used as a regularization tool. This benefits from the smoothness criterion in Dirichlet energy: it measures the “roughness” or “degree of variation” of a function by integrating the square of its gradient. When the Dirichlet energy of a function $\textrm{x}$ is small, it means the gradient of the function is relatively small across the entire region, indicating smooth changes and good smoothness. However, excessive smoothness may also lead to the homogenization of node features, losing their discriminative power. To prevent this over-smoothing phenomenon, energy GNN^32^ cleverly sets lower and upper bounds on the Dirichlet energy at each layer to avoid performance degradation due to excessive smoothness. Recently, the DESAlign^33^ method uses the generalizable theoretical principles inspired by Dirichlet energy to guide multi-modal knowledge graph learning, ensuring the optimization of semantic consistency. Although Dirichlet energy has shown great potential in these applications, it still has some issues. For example, it overly relies on the accuracy of the initial graph structure and tends to ignore the fidelity of observed data. If the initial graph structure itself has missing or erroneous information, relying solely on Dirichlet energy for optimization may lead to results that increasingly deviate from the true situation. Additionally, pursuing smoothness alone may overly suppress local differences in node features, causing the completion results to significantly deviate from the real observed data.

## Methodology

**Fig. 1:**
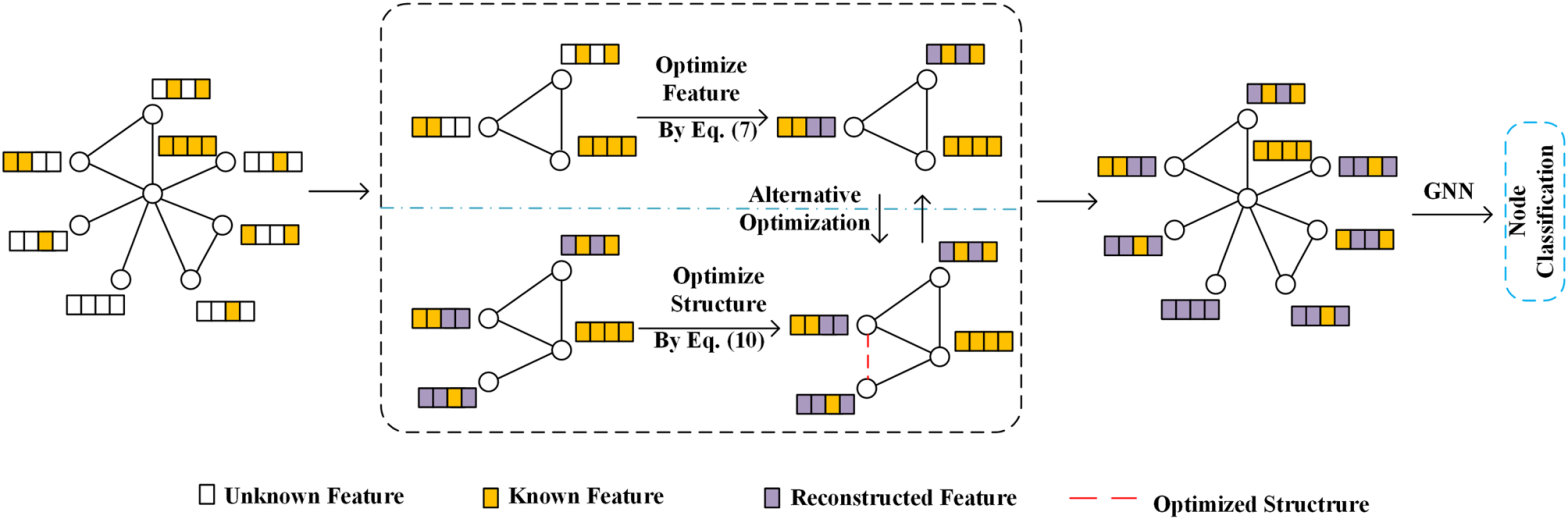
The whole framework of the proposed EGS method.

In this section, we will detail the proposed EGS framework. Figure 1 visually shows the overall architecture of our method. For data with missing attributes, especially missing node features, the goal of this framework is to learn an optimal graph representation by dynamically updating the graph structure after an initial attribute reconstruction. This helps explore higher-order relationships in the data and addresses the missing attribute problem from two perspectives. Specifically, our method first constructs a graph to represent the correlations between data points. During this process, the framework flexibly supports any graph construction strategy. Based on the initial graph, to overcome the limitations of Dirichlet energy–its over-reliance on the accuracy of the initial graph structure and potential neglect of observed data fidelity–, we build an objective function that jointly optimizes attribute features and graph structure. In each iteration, starting from the attribute level, we first reconstruct the feature matrix. Then, based on the updated feature matrix, we further update the graph structure to obtain the updated normalized Laplacian matrix. This process repeats until the objective function value stabilizes and no longer decreases significantly. During this time, the graph structure is continuously optimized. Finally, through the multi-regularization joint optimization strategy, we combine prior knowledge with data-driven learning to reconstruct an optimal graph representation. This lays a solid foundation for subsequent classification tasks. The following sections will provide a more detailed analysis of this method.

### EGS problem formulation

In this study, we consider an undirected graph defined by an adjacency matrix $\textrm{A}$ and a node feature matrix $\textrm{X}$. The normalized Laplacian matrix of the graph, $\Delta =\textrm{I}-\widetilde{\textrm{A}}$, is a key component of a positive semi-definite matrix. It is made up of the normalized adjacency matrix $\widetilde{\textrm{A}}$ and the degree matrix $\textrm{D}$. Specifically, $\widetilde{\textrm{A}}=\textrm{D}^{-\frac{1}{2}} \textrm{AD}^{-\frac{1}{2}}$ is obtained by multiplying the diagonal matrix $\textrm{D}$ by $\textrm{A}$. The degree matrix $\textrm{D}=\operatorname {diag}\left( \sum _{\textrm{j}} \textrm{a}_{1 \textrm{j}}, \ldots , \sum _{\textrm{j}} \textrm{a}_{\textrm{nj}}\right)$ contains the degree information of each node.

In graph learning with missing features, the structure of graph $\textrm{G}$ is typically assumed to be known, while labels and node features are only partially available on a subset of nodes. However, missing severe node features can alter the semantic information of the graph, leading to changes in the graph structure. For example, in molecular graphs, missing nodes can change molecular properties, resulting in entirely different molecules. Therefore, updating the graph structure plays a crucial role in addressing feature-missing problems. Specifically, our goal is to learn a function $\ell (\textrm{X},\Delta ,\textrm{G})$ that infers missing feature values using the existing graph structure, dynamically updates the graph structure via the standard Laplacian matrix, and ultimately reconstructs the complete feature vector $\textrm{X}$ from the known partial features $\mathrm {X_{k}}$. To define this objective function, two fundamental conditions must be satisfied.

Smoothness Constraint: The imputed features should exhibit smoothness in both attributes and structure. Specifically, this requires the feature matrix to be smooth on the normalized Laplacian matrix. Dirichlet energy enforces the smoothness of the imputed feature matrix across the graph structure by penalizing differences between neighboring node features, aligning with the homophily assumption of graph data (neighboring nodes share similar features). Additionally, the Laplacian matrix directly encodes the graph’s topological structure, incorporating structural information into the optimization objective through a quadratic form, thereby preventing imputation results from contradicting the graph topology. To this end, this paper adopts Dirichlet energy as a quantitative measure of smoothness. The mathematical expression of the Dirichlet energy $\textrm{f}$ is shown in Eq. (1):

1 $$\begin{aligned} \textrm{f}=\frac{1}{2} \textrm{X}^{\textrm{T}} \Delta \textrm{X}=\frac{1}{2} \sum _{\textrm{ij}} \tilde{\textrm{a}}_{\textrm{ij}}\left( \textrm{X}_{\textrm{i}}-\textrm{X}_{\textrm{j}}\right) ^{2} \end{aligned}$$

where $\textrm{X}$ represents the feature matrix. $\Delta$ is the normalized Laplacian matrix. $\mathrm {X_{i}}$ and $\tilde{\textrm{a}}_{\textrm{ij}}$ are individual elements in the feature matrix $\textrm{X}$ and the normalized adjacency matrix $\tilde{\textrm{A}}$.

Empirical Loss Minimization: The original Dirichlet energy may overlook the fidelity of observed data. Relying solely on smoothness could excessively suppress local differences in node features, causing imputation results to deviate from the true observed data $\mathrm {X_{0}}$ (e.g., some nodes already have high-confidence labels). By using the Frobenius norm, the imputed feature matrix $\textrm{X}$ is forced to remain as close as possible to the initial observed data $\mathrm {X_{0}}$, preventing the loss of known information due to over-smoothing. Unlike node-wise L2 constraints, the Frobenius norm globally constrains the difference between $\textrm{X}$ and $\mathrm {X_{0}}$, making it more suitable for matrix-based feature imputation. Additionally, the original Dirichlet energy overly relies on the accuracy of the initial graph structure. When optimizing $\Delta$, its deviation from the initial structure $\Delta _{0}$ is constrained to prevent instability in structure learning caused by data noise while also reducing dependence on the initial graph structure during later optimization. Therefore, this paper incorporates the Frobenius norm into the objective function to quantify the difference between the transformed feature matrix and the initial feature matrix, as well as the deviation between the transformed normalized Laplacian matrix and the initial normalized Laplacian matrix, ensuring better smoothing of attributes and graph structure. The specific expressions are shown in Eqs. (2) and (3):

2 $$\begin{aligned} & \mathscr {R}_{\text{ emp1 }}=\left\| \textrm{X}-\textrm{X}_{0}\right\| _{\textrm{F}}^{2} \end{aligned}$$

3 $$\begin{aligned} & \mathscr {R}_{\textrm{emp2}}=\left\| \Delta -\Delta _{0}\right\| _{\textrm{F}}^{2} \end{aligned}$$

where the $\mathrm {X_{0}}$ and $\Delta _{0}$ are present in the initial feature matrix and the initial normalized Laplacian matrix, respectively. $\mathscr {R}_{\text{ emp1 }}$ and $\mathscr {R}_{\text{ emp2 }}$ denotes variance.

Combining the above two conditions, the objective function for multi-objective optimization is constructed using two Frobenius norm regularizations and Dirichlet energy. The convexity of the Dirichlet energy and the Frobenius norm ensures that the objective function converges to a local optimal solution during alternating optimization. This approach not only effectively addresses overfitting but also simplifies mathematical processing. The expression is shown in Eq. (4):

4 $$\begin{aligned} \ell (\textrm{X}, \Delta , \textrm{G}) = \frac{1}{2} \textrm{X}^{\textrm{T}} \Delta \textrm{X} + \lambda _{1}\left\| \textrm{X}-\textrm{X}_{0}\right\| _{\textrm{F}}^{2} \! + \lambda _{2}\left\| \Delta -\Delta _{0}\right\| _{\textrm{F}}^{2} \end{aligned}$$

where the $\lambda _{1}$ and $\lambda _{2}$ are the parameters used to balance the weights of the components in the objective function. By minimizing this function, we dynamically address both attribute reconstruction and graph structure updating, achieving imputed features that satisfy smoothness requirements while remaining consistent with the initial data.

### Optimized solution

In this section, we detail the optimization of the objective function described in Eq. (4), which involves the joint learning of the feature matrix and the normalized Laplacian matrix. To achieve this, we employ the Alternating Direction Method of Multipliers, dynamically optimizing by fixing one variable and updating the other, iterating until the objective function converges or improvements become negligible. This indicates that the function’s gradient remains relatively small across the domain, and the function varies smoothly. Such enhanced smoothness facilitates gradual convergence toward the optimal solution.

Specifically, we first perform attribute reconstruction by optimizing the feature matrix $\textrm{X}$ using the existing normalized Laplacian matrix $\Delta$. After fixing $\Delta$, the subproblem for optimizing $\textrm{X}$ can be expressed as Eq. (5):

5 $$\begin{aligned} \arg \min _{\textrm{X}} \ell (\textrm{X}, \textrm{G})=\frac{1}{2} \textrm{X}^{\textrm{T}} \Delta \textrm{X}+\lambda _{1}\left\| \textrm{X}-\textrm{X}_{0}\right\| _{\textrm{F}}^{2} \end{aligned}$$

At this point, the function is convex. To solve this optimization problem, we compute the gradient of Eq. (5) concerning $\textrm{X}$, yielding the result expressed in Eq. (6):

6 $$\begin{aligned} \nabla _{\textrm{X}} \ell =\Delta \textrm{X}+2 \lambda _{1}\left( \textrm{X}-\textrm{X}_{0}\right) \end{aligned}$$

Setting the gradient to zero to find the extremum point yields Eq.(7). Since $\Delta$ is the normalized Laplacian matrix and is positive semi-definite, we prove that $(\frac{1}{2\lambda _{1} } \Delta + \textrm{I})$ is invertible (see Proof of Reversibility for details). The iterative equation for updating the feature matrix is shown in Eq. (7):

7 $$\begin{aligned} \textrm{X}=\left( \frac{1}{2 \lambda _{1}} \Delta +\textrm{I}\right) ^{-1} \textrm{X}_{0} \end{aligned}$$

Next, we update the graph structure by optimizing the normalized Laplacian matrix $\Delta$ given the feature matrix $\textrm{X}$. After fixing $\textrm{X}$, the subproblem for optimizing $\Delta$ is shown in Eq. (8):

8 $$\begin{aligned} \arg \min _{\Delta } \ell (\Delta , \textrm{G})=\frac{1}{2} \textrm{X}^{\textrm{T}} \Delta \textrm{X}+\lambda _{2}\left\| \Delta -\Delta _{0}\right\| _{\textrm{F}}^{2} \end{aligned}$$

Similarly, the function is convex at this point. Computing the gradient of Eq. (8) concerning $\Delta$ yields the result expressed in Eq. (9):

9 $$\begin{aligned} \nabla _{\Delta } \ell =\frac{1}{2} \textrm{X}\textrm{X}^{\textrm{T}}+2 \lambda _{2}\left( \Delta -\Delta _{0}\right) \end{aligned}$$

By setting the derivative equal to zero to find the extremum point, it is straightforward to prove that $\frac{1}{4\lambda _{2} }\textrm{I}$ is invertible. Accordingly, we derive the iterative equation shown in Eq. (10) to update the normalized Laplacian matrix:

10 $$\begin{aligned} \Delta =\Delta _{0}-\frac{1}{4 \lambda _{2}} \textrm{XX}^{\textrm{T}} \end{aligned}$$

The alternating optimization of the two forms a closed-loop feedback mechanism, effectively enhancing the rationality of the imputation results. In alternating optimization, each subproblem is convex, ensuring convergence to a local optimum in every update step. Through iterative refinement, the system progressively approaches the global optimal solution.

**Figure:**
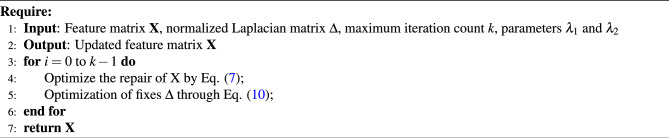
Algorithm 1 Outlines the implementation details of this alternating optimization process.

### Computation complexity

In this section, we analyze the computational complexity of our proposed EGS method. The EGS method mainly consists of two alternating optimization steps: updating the feature matrix $\textrm{X}$ and updating the normalized Laplacian matrix $\Delta$.

- Feature Matrix Optimization: The feature matrix optimization step involves solving a linear system of equations. The complexity of inverting a matrix is generally $O(N^3)$;
- Graph Structure Update: The update of the normalized Laplacian matrix $\Delta$. The complexity of this step is dominated by the matrix multiplication, which is $O(Nd^2)$.

## Experiments

### Experimental settings

#### Datasets

To demonstrate the proposed EGS’s effectiveness and scalability, we conduct extensive experiments on five datasets, including the citation networks dataset^34^ (Cora, Citeseer and Pubmed) and the Amazon dataset^35^ (Amazon-Photo and Amazon-Computers). To more clearly demonstrate the characteristics of these datasets, we denote the Amazon-Photo dataset as Photo and the Amazon-Computers dataset as Computers. We report the relevant statistics in detail in Table 1, including the number of nodes, the number of edges, the feature dimensions, and the number of categories.

**Table 1:** Details of the datasets.

Dataset	Nodes	Edges	Features	Classes
Cora	2, 485	5, 069	1, 433	7
Citeseer	2, 120	3, 679	3, 703	6
Pubmed	19, 717	44, 324	500	3
Photo	7, 487	119, 043	745	8
Computers	13, 381	245, 778	767	10

#### Implementation details

This study adopts the following setup to ensure the reliability and reproducibility of the results. Five datasets mentioned in Section Datasets are used for random but fixed-ratio training/ validation/ testing splits, and different missing feature masks are applied for each experimental run (unless specified differently in specific codes or papers). Specifically, the experiments followed the strategy of a previous study^36^, allocating 20 nodes per class for the training set, 1500 nodes in total for the validation set, and the rest for the test set.

Regarding hyperparameter selection, two strategies have been employed to ensure a fair comparison with existing methods. Where hyperparameters are delineated in the original papers or codes, they have been directly used for the reproduction and subsequent comparison of results. For methods without provided parameters, we employ the same hyperparameter configurations within this paper to facilitate replication and comparative analysis.

During model training, we employed the Adam optimizer with a learning rate of 0.001. For the graph neural networks, two-layer and four-layer architectures were implemented, with a maximum training epoch of 500. To prevent overfitting, we incorporated early stopping (triggered after 10,000 consecutive steps without validation loss improvement) and a dropout rate of 0.9. Empirical results demonstrate full convergence across all experimental datasets within 35 iterations. To assess model performance, we identify three pivotal parameters: $\lambda _{1}$, $\lambda _{2}$ and the hidden dimension, which are subjected to a grid search. A detailed examination of the parameter selection process and its influence on model performance is reserved for the ensuing section on parameter analysis (Section Effects of Parameter). All experiments are conducted on the RTX 3080x2 (20GB) and the RTX 4090 (24GB), ensuring sufficient computational resources and accelerating execution speed.

#### Comparative experiment setting

To comprehensively evaluate the performance of EGS in addressing missing attributes for node classification tasks, we conduct comparative experiments against existing missing attribute handling methods. In this section, we present the names and configurations of the selected comparison methods. Detailed experimental results and conclusions will be provided in Section Experiment results.

The specific comparison methods include partial GNN (PaGNN)^37^, GCN for missing features (GCNMF)^38^, FP, pseudo-confidence-based feature imputation (PCFI)^39^, a dual-channel GNN (D2PT)^40^ and the topology-driven attribute recovery (TDAR)^41^. For GCNMF and PaGNN, due to the absence of an official implementation, our replication and comparative analysis are conducted based on the implementation specifics delineated in the FP paper and its corresponding code. For FP, PCFI and TDAR, the experiments are executed utilizing their publicly available code and hyperparameters, with only the missing rate being adjusted for replication purposes. Given that D2PT does not provide sufficient settings for the Photo and Computers datasets, we confine our comparative experiments using D2PT to the Cora, Citeseer and Pubmed datasets.

Moreover, we compare our approach with traditional simple interpolation methods^42^, such as setting missing features to zero or random values drawn from a standard Gaussian distribution. Additionally, we benchmark our method against other commonly used computational-based approaches, including variational auto-encoder^43^, missing data imputation using generative adversarial nets^44^, or SAT, noting that prior research has indicated their generally lower performance compared to GCNMF and PaGNN in handling graph data.

### Experiment results

Table 2 presents a performance comparison between the EGS method and six other mainstream approaches, focusing on node classification accuracy and standard deviation across different missing rates for five datasets. The detailed analysis leads to the following conclusions:

**Table 2:** Thenode classification accuracy on Cora, Citeseer, Pubmed, Photo and Computers datasets. Bold data indicates suboptimal values of accuracy, and bold and enlarged font indicates optimal values of accuracy. (Evaluation criteria: Accuracy ± Standard deviation)

Dataset	Method	Missing rate (%)				
		5	15	35	55	75
Cora	PAGNN (arXiv 2020)^37^	81.36 ± 1.32	80.61 ± 1.55	78.96 ± 1.81	77.52 ± 1.18	74.05 ± 1.66
	GCNMF (FGCS 2021)^38^	81.06 ± 1.64	80.55 ± 1.22	79.27 ± 1.51	74.84 ± 1.31	68.28 ± 1.38
	FP (LoG 2022)^13^	80.06 ± 0.79	78.78 ± 1.00	76.67 ± 1.60	76.89 ± 0.33	77.36 ± 1.26
	PCFI (ICLR 2023)^39^	81.34 ± 1.50	**81.56 ± 1.60**	80.41 ± 1.75	79.98 ± 1.64	79.88 ± 1.92
	D2PT (KDD 2023)^40^	76.64 ± 2.52	76.22 ± 2.69	75.79 ± 2.47	75.29 ± 2.88	73.97 ± 2.65
	TDAR(IoT 2025)^41^	**83.01 ± 3.26**	**81.56 ± 2.73**	**84.17 ± 2.84**	**83.62 ± 1.26**	${{\textbf {82.37}}\pm {\textbf {1.75}}}$
	EGS (ours)	${{\textbf {84.77}}\pm {\textbf {1.12}}}$	${{\textbf {84.57}}\pm {\textbf {0.81}}}$	${\textbf {84.48}}\pm {\textbf {0.90}}$	${\textbf {83.95}}\pm {\textbf {0.64}}$	**82.13 ± 0.75**
Citeseer	PAGNN (arXiv 2020)^37^	68.26 ± 1.07	66.84 ± 0.62	65.77 ± 1.71	65.19 ± 1.06	61.90 ± 1.98
	GCNMF (FGCS 2021)^38^	67.39 ± 1.20	66.68 ± 1.31	66.06 ± 1.96	63.71 ± 1.84	59.77 ± 3.15
	FP (LoG 2022)^13^	**69.97 ± 1.42**	68.97 ± 1.01	67.55 ± 1.70	66.35 ± 1.93	65.55 ± 2.11
	PCFI (ICLR 2023)^39^	69.32 ± 1.36	**69.55 ± 1.23**	**68.81 ± 1.31**	**67.77 ± 1.48**	**66.97 ± 1.38**
	D2PT (KDD 2023)^40^	65.58 ± 2.06	64.80 ± 1.35	64.06 ± 1.91	62.74 ± 1.31	57.60 ± 2.08
	TDAR(IoT 2025)^41^	65.93 ± 1.03	65.60 ± 0.82	65.66 ± 1.94	66.55 ± 0.72	62.86 ± 1.83
	EGS (ours)	${{\textbf {70.96}}\pm {\textbf {1.36}}}$	${\textbf {70.64}}\pm {\textbf {1.73}}$	${\textbf {70.48}}\pm {\textbf {1.64}}$	${\textbf {68.97}}\pm {\textbf {1.28}}$	${\textbf {67.41}}\pm {\textbf {1.09}}$
Pubmed	PAGNN (arXiv 2020)^37^	75.92 ± 1.81	75.44 ± 4.82	74.53 ± 2.13	73.62 ± 1.42	71.65 ± 2.17
	GCNMF (FGCS 2021)^38^	74.28 ± 1.18	73.15 ± 1.83	71.65 ± 1.97	68.19 ± 1.56	61.24 ± 1.59
	FP (LoG 2022)^13^	76.41 ± 0.64	75.61 ± 1.12	75.35 ± 0.70	74.55 ± 1.96	73.59 ± 2.83
	PCFI (ICLR 2023)^39^	76.72 ± 2.02	75.89 ± 2.00	75.18 ± 1.79	74.72 ± 1.65	73.96 ± 2.10
	D2PT (KDD 2023)^40^	70.66 ± 3.07	70.23 ± 1.60	70.19 ± 2.76	69.03 ± 2.23	68.63 ± 3.40
	TDAR(IoT 2025)^41^	**77.07 ± 3.94**	**77.99 ± 2.83**	${\textbf {80.06}}\pm {\textbf {4.92}}$	${\textbf {80.35}}\pm {\textbf {3.97}}$	${\textbf {80.59}}\pm {\textbf {3.86}}$
	EGS (ours)	${\textbf {80.37}}\pm {\textbf {0.90}}$	${\textbf {79.79}}\pm {\textbf {0.83}}$	**79.34 ± 1.53**	**78.95 ± 1.24**	**77.01 ± 1.80**
Photo	PAGNN (arXiv 2020)^37^	84.58 ± 1.96	84.40 ± 1.10	84.02 ± 1.04	83.35 ± 1.48	82.91 ± 1.96
	GCNMF (FGCS 2021)^38^	83.99 ± 1.05	83.32 ± 1.55	78.50 ± 1.43	70.29 ± 2.84	62.68 ± 4.17
	FP (LoG 2022)^13^	91.35 ± 0.85	91.13 ± 1.06	90.78 ± 1.00	90.05 ± 1.03	89.09 ± 1.44
	PCFI (ICLR 2023)^39^	89.88 ± 1.28	89.22 ± 1.27	88.35 ± 1.40	87.39 ± 1.79	86.69 ± 1.65
	TDAR(IoT 2025)^41^	**90.59 ± 2.03**	**92.33 ± 1.28**	**92.30 ± 1.02**	**92.49 ± 1.84**	${\textbf {92.07}}\pm {\textbf {2.41}}$
	EGS (ours)	${\textbf {92.89}}\pm {\textbf {1.09}}$	${\textbf {92.76}}\pm {\textbf {1.16}}$	${\textbf {92.63}}\pm {\textbf {1.55}}$	${\textbf {92.53}}\pm {\textbf {1.52}}$	**91.64 ± 1.68**
Computers	PAGNN (arXiv 2020)^37^	82.99 ± 2.35	82.84 ± 3.25	82.79 ± 1.80	82.12 ± 3.42	81.66 ± 1.55
	GCNMF (FGCS 2021)^38^	81.80 ± 2.06	79.12 ± 1.17	75.59 ± 1.93	70.29 ± 1.26	62.68 ± 3.12
	FP (LoG 2022)^13^	84.32 ± 0.72	84.12 ± 4.24	83.48 ± 2.52	83.42 ± 1.77	82.54 ± 2.43
	PCFI (ICLR 2023)^39^	83.32 ± 1.65	81.35 ± 1.14	79.01 ± 1.78	77.33 ± 1.96	76.49 ± 1.55
	TDAR(IoT 2025)^41^	${\textbf {87.20}}\pm {\textbf {2.32}}$	${\textbf {90.06}}\pm {\textbf {1.72}}$	${\textbf {90.27}}\pm {\textbf {0.96}}$	${\textbf {89.92}}\pm {\textbf {3.52}}$	${\textbf {89.80}}\pm {\textbf {2.47}}$
	EGS (ours)	**86.93 ± 0.48**	**86.66 ± 1.59**	**86.32 ± 0.88**	**86.14 ± 1.33**	**85.87 ± 1.13**

- The EGS method significantly improves the performance of the downstream GCN model by filling in missing features during the preprocessing stage. In the vast majority of missing cases, EGS achieves higher classification accuracy and more stable standard deviation than other methods, demonstrating its effectiveness in handling missing attribute problems.
- Experiment results on five benchmark datasets show that the EGS method consistently outperforms PAGNN, GCNMF, FP, PCFI and D2PT in performance. In terms of accuracy, a key metric for model evaluation, EGS demonstrates a clear advantage. Specifically, under five missing rates, the average accuracy of EGS exceeds that of PAGNN, GCNMF, FP, PCFI and D2PT by $5.39\%$, $10.15\%$, $3.37\%$, $3.88\%$ and $8.15\%$, respectively. Notably, even compared to the recently popular PCFI and D2PT, EGS achieves significant gains, with maximum improvements of $9.81\%$–$9.83\%$, respectively. This indicates that although D2PT handles complex missing patterns and interactions well, and PCFI shows relative robustness under extremely high missing rates, both methods still lag behind EGS in accuracy when the missing rate ranges from $5\%$–$75\%$. In terms of robustness, measured by standard deviation, EGS also shows superiority. Its average stability improves by 0.64, 0.60, 0.29, 0.40 and 1.15 compared to PAGNN, GCNMF, FP, PCFI and D2PT, respectively. These results validate the effectiveness of the alternating optimization strategy used in EGS between the graph structure and the feature matrix. This strategy better captures the complex intrinsic relationships in data. Overall, the findings not only confirm the strong performance of EGS but also highlight the crucial role of graph structure optimization in enhancing feature learning.
- In a systematic comparison with the state-of-the-art method TDAR, the EGS method further demonstrates its superior performance. Across five benchmark datasets and five missing rates, EGS achieves an average accuracy improvement of $0.55\%$ over TDAR. Specifically, on the Citeseer dataset, EGS outperforms TDAR by an average of $4.37\%$. On the Cora and Photo datasets, under low to moderate missing rates ($5\%$–$15\%$), EGS consistently achieves higher node classification accuracy, with a maximum improvement of $3.01\%$. Within the $35\%$–$55\%$ missing rate range, although the improvement margin of EGS is relatively limited, its performance still provides strong validation for the effectiveness of the method. EGS is only slightly outperformed by TDAR under the high $75\%$ missing rate. However, such high missing rates are rare in real-world scenarios, making EGS’s overall accuracy advantage more meaningfully under typical conditions. On the Pubmed dataset, EGS surpasses TDAR by an average of $2.55\%$ at low missing rates ($5\%$, $15\%$). While EGS shows improvements, TDAR achieves better accuracy under high missing rates on the Pubmed dataset and across all missing rates on the Computers dataset. This can be attributed to dataset-specific characteristics and the design of TDAR. For example, Pubmed contains structural noise due to interdisciplinary citations, and Computers has large-scale and high-dimensional features. TDAR’s adoption of node homogeneity score and non-linkage similarity calibration helps address these challenges effectively. In terms of robustness, EGS outperforms TDAR on all five datasets, reducing the average standard deviation by 1.08, indicating better result stability. In summary, although TDAR demonstrates certain advantages in specific datasets and extreme missing rate conditions, EGS exhibits more robust generalization and stability across a wide range of benchmark datasets, typical missing rate intervals, and overall performance metrics.

### Ablation studies

#### Effects of different regularization terms

To validate the effectiveness of EGS’s design principles, we conducted comprehensive ablation studies on the Cora and Pubmed datasets. The experiments were designed to isolate components of the objective function, focusing on the impact of regularization terms. When retaining only the attribute regularization term (EGS (w/o $\lambda _{2}$)), the model inferred missing attributes using existing keywords but failed to effectively link thematically related paper nodes without citation relationships due to a lack of structural constraints. When retaining only the structural regularization term (EGS (w/o $\lambda _{1}$)), the model enhanced latent thematic associations but risked over-connecting nodes sharing keywords without direct citations, introducing noisy edges. Removing all regularization terms (EGS (w/o all)) degraded collaborative attribute-structure optimization, leaving only the Dirichlet energy baseline. In contrast, the full EGS model with dual regularization terms balanced attribute completion and structural refinement through coordinated $\lambda _{1}$ and $\lambda _{2}$ optimization. For fairness, all experiments maintained parameter consistency with the original EGS configuration.

The ablation study results detailed in Table 3 demonstrate that EGS significantly outperforms methods with single regularization terms in node classification, achieving improvements of $1.3\%$ – $4.09\%$. Compared to methods without regularization, the improvements further increase to $3.54\%$ – $4.62\%$. The experiments indicate that incorporating both attribute and structural regularization into the Dirichlet energy framework prevents over-smoothing. This ensures the completion process is not overwhelmed by neighborhood features. Furthermore, the completed attributes help identify latent similar nodes, correcting structural sparsity through optimized connections. Notably, these improvements become more pronounced as the missing rate increases. These findings strongly support the critical role of dual regularization in enhancing EGS’s classification performance.

**Table 3:** Comparison results with different regularization terms. Data in bold indicates optimal values.

Dataset	Case	5%	15%	35%	55%	75%
Cora	EGS (w/o all)	81.91	81.03	80.58	79.58	78.06
	EGS (w/o $\lambda _{2}$)	83.47	83.01	81.66	81.02	79.81
	EGS (w/o $\lambda _{1}$)	82.50	81.64	81.52	80.42	79.03
	EGS (ours)	**84.77**	**84.57**	**84.48**	**83.95**	**82.13**
Pubmed	EGS (w/o all)	75.95	75.83	75.29	74.33	73.21
	EGS (w/o $\lambda _{2}$)	78.17	77.91	76.56	74.86	73.68
	EGS (w/o $\lambda _{1}$)	76.75	76.02	75.72	74.78	73.58
	EGS (ours)	**80.37**	**79.79**	**79.34**	**78.95**	**77.01**

#### Effects of different optimization methods

To systematically evaluate the effects of optimization strategies on attribute-incomplete graphs, we performed comprehensive experiments on the Cora and Pubmed benchmarks, conducting empirical comparisons between joint regularization optimization and our proposed alternating optimization paradigm. The scenario where both regularization terms were optimized simultaneously was labeled as “EGS-si,” and the other, which involves the alternating optimization of the two regularization terms as mentioned in this paper, was labeled as “EGS” To ensure the fairness of the experiments, we used the EGS method to maintain consistency in all experimental parameters as much as possible. The results of the experiments are shown in Table 4. The experimental results indicate that the alternating optimization method outperforms the simultaneous optimization method on all datasets. Specifically, the alternating optimization method improved the accuracy in node classification tasks by $0.9\%$−$2.92\%$ and demonstrated better stability when dealing with high rates of missing data.

**Table 4:** Comparison results with different optimization methods. Data in bold indicates optimal values.

Dataset	Case	5%	15%	35%	55%	75%
Cora	EGS-si	83.87	82.78	82.23	81.62	80.33
	EGS (ours)	**84.77**	**84.57**	**84.48**	**83.95**	**82.13**
Pubmed	EGS-si	78.55	77.85	76.56	76.03	75.23
	EGS (ours)	**80.37**	**79.79**	**79.34**	**78.95**	**77.01**

bold data directly indicatesoptimal values of accuracy

#### Effects of parameter

In our proposed EGS, three parameters are introduced: $\lambda _{1}$, $\lambda _{2}$, and the number of hidden layers *H*. The parameters $\lambda _{1}$ and $\lambda _{2}$ are employed to balance the dual constraints concerning nodes’ structure relationships updating, and attribute reconstruction, respectively. The parameter *H* regulates the number of hidden layers within the GCN. To assess the impact of different parameter selections on the node classification results, we conduct experiments on the Cora and Pubmed datasets in the data missing rate of $5\%$.

**Fig. 2:**
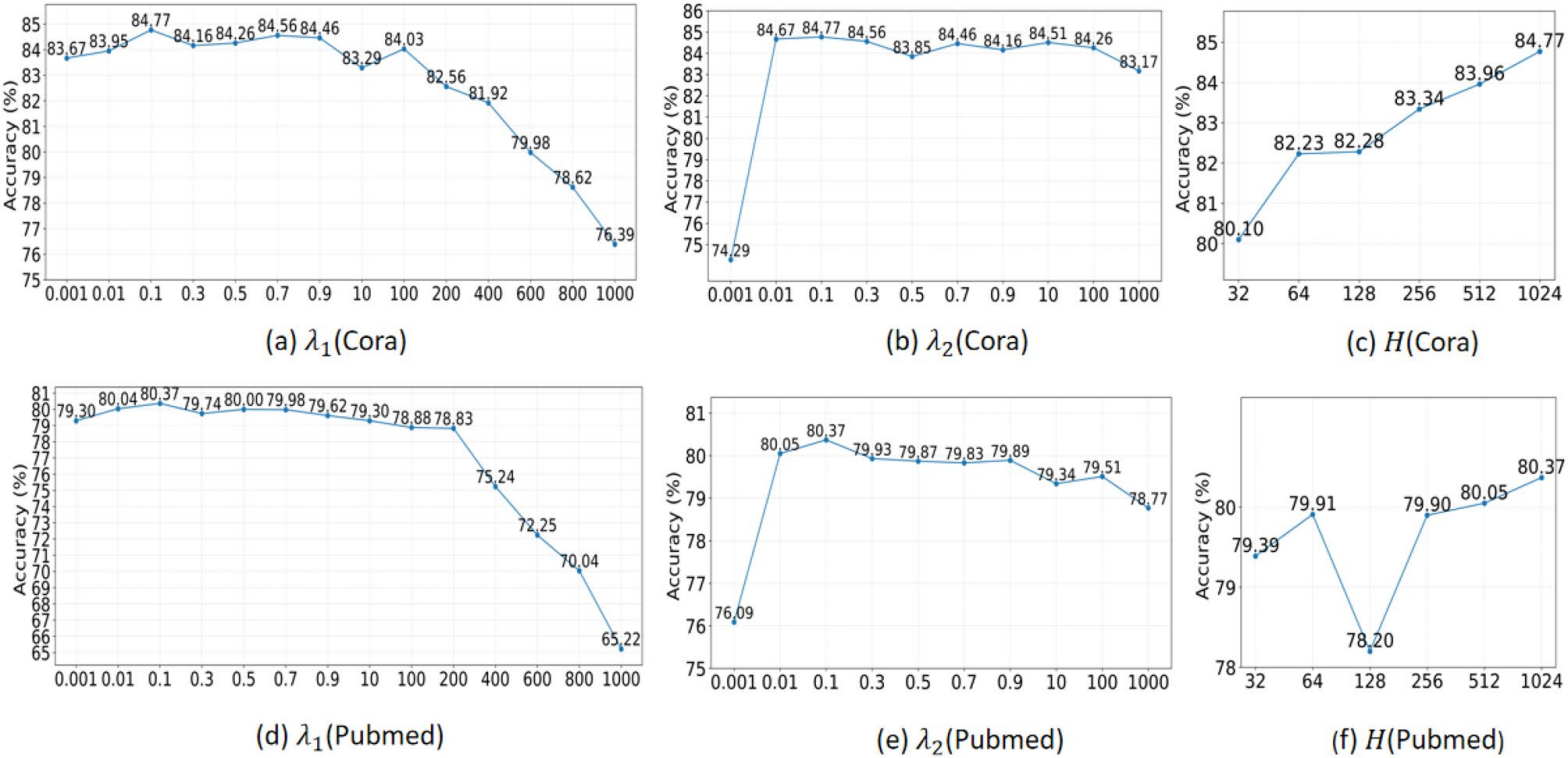
Comparison results with different hyperparameters on the Cora and Pubmed datasets.

The results as shown in Fig. 2. Specifically, Fig. 2a–c show the effects of different $\lambda _{1}$, $\lambda _{2}$ and *H* on the Cora dataset, respectively. Figure 2d–e show the effects on the Pubmed dataset. The node classification accuracy increased with $\lambda _{1}$ and $\lambda _{2}$ values up to 0.1, peaked at 0.1, and stabilized thereafter. Significant performance degration for EGS occurred only when $\lambda _{1} > 100$, indicating strong robustness of the attribute reconstruction regularization term. For $\lambda _{2}$, the model showed high parameter sensitivity at $\lambda _{2} < 0.01$. Performance degradation emerged only when $\lambda _{2} > 100$, suggesting that excessive structural constraints introduce bias. Notably, accuracy remained stable within $\lambda _{2} \in [0.01, 100]$, demonstrating operational stability in this range. Based on these findings, we set $\lambda _{1}$ = 0.1, $\lambda _{2}$ = 0.1 and $H = 1024$ as optimal configurations.

#### Effects of different variants

To validate the applicability and generalization of the EGS method, we conduct a series of ablation experiments. These studies integrate traditional multilayer perceptron (MLP)^45^ with popular GNN variants like GCN, GraphSAGE, simplifying GCN (SGC)^46^ and graph attention networks (GAT)^47^, explore combinations with methods such as GCNMF and PAGNN.

We performed experiments on well-known datasets, Cora and Pubmed and evaluated these methods in the missing rate of $5\%$. To ensure fairness, we use the published optimal parameters for the comparative FP method. The accuracies of different methods combined with various variants in node classification tasks are shown in Table 5. The table data shows that EGS outperforms FP in most variants, providing evidence for the versatility and extensiveness of the EGS approach. Notably, while both EGS and FP are compatible with various GNN architectures and EGS shows superior overall performance, its performance on MLP is significantly weaker than other models. This can be attributed to two factors. First, MLP relies solely on node features and completely ignores graph structural information. Consequently, it cannot leverage the structure-feature co-representation optimized by EGS through Dirichlet energy. Second, EGS generates highly smoothed, low-variance features by enforcing feature homogeneity among neighboring nodes via Dirichlet energy. However, MLP depends on nonlinear discriminative feature patterns for classification. This smoothing effect diminishes the separability of features, directly weakening MLP’s discriminative capability.

**Table 5:** Comparison of different variants in the missing rate of 5$\%$. Data in bold indicates optimal values.

Dataset	Method	MLP	SGC	GraphSAGE	GCN	GCNMF	PAGNN	GAT
Cora	FP (LoG 2022)^14^	47.35	81.79	80.51	83.27	81.97	81.34	80.59
	EGS (ours)	**55.53**	**83.14**	**81.69**	**84.77**	**82.64**	**82.38**	**82.33**
Pubmed	FP (LoG 2022)^14^	63.89	74.10	74.23	75.64	74.61	75.74	77.06
	EGS (ours)	**66.90**	**76.46**	**77.21**	**80.37**	**78.43**	**79.30**	**77.95**

#### Ablation studies under high missing rate scenarios

To verify the robustness boundaries of EGS in extreme data missing scenarios, we conducted experiments with $85\%$–$95\%$ missing rates on the Citeseer dataset. As shown in Table 6, at $85\%$ missing rate, EGS significantly outperformed traditional graph learning methods (e.g., GCNMF, PAGNN) and emerging attribute-missing models (e.g., D2PT, TDAR), achieving up to $15.66\%$ accuracy improvement over baselines. EGS also achieved comparable performance to PFCI and FP, which excel in high missing rate scenarios. However, at a $95\%$ missing rate, EGS exhibited a noticeable accuracy decline and underperformed PFCI and FP.

This phenomenon is directly linked to EGS’s algorithmic design. By introducing Dirichlet energy constraints and dual regularization terms, EGS explicitly models implicit structure-feature relationships. This mechanism effectively captures the intrinsic data manifold at regular missing rates ($\le 75\%$), where its standard deviations are significantly lower than competitors’, demonstrating stability advantages. When missing rates exceed $85\%$, severe disconnections occur between topological information and attribute distributions, invalidating the local smoothness assumption in Dirichlet energy constraints. This weakens the regularization terms’ ability to compensate for missing patterns. Notably, extreme missing rates ($>85\%$) occur far less frequently in real-world scenarios. EGS has already demonstrated generalizability advantages within regular missing rate ranges (see Table 2). Future work will focus on adaptive energy constraint functions to enhance robustness against topological disconnections in extreme missing scenarios.

**Table 6:** The performance of node classification under $85\%$ and $95\%$ missing rate. Bold data indicates suboptimal values of accuracy, and bold and enlarged font indicates optimal values of accuracy. (Evaluation criteria: Accuracy ± Standard deviation)

Dataset	Method	Missing rate	
		85%	95%
Citeseer	PAGNN (arXiv 2020)^38^	58.74 ± 2.41	53.61 ± 3.28
	GCNMF (FGCS 2021)^39^	57.39 ± 2.00	50.81 ± 1.96
	FP (LoG 2022)^14^	65.52 ± 2.04	**65.87 ± 1.28**
	PCFI (ICLR 2023)^40^	${\textbf {66.63}}\pm {\textbf {1.79}}$	${\textbf {66.10}}\pm {\textbf {1.84}}$
	D2PT (KDD 2023)^41^	53.46 ± 2.10	43.32 ± 3.26
	TDAR(arXiv 2025)^42^	54.39 ± 2.63	53.74 ± 5.58
	EGS (ours)	**65.89 ± 2.43**	58.98 ± 1.24

## Conclusion

This paper proposes a general solution strategy, EGS, for handling missing attributes in graph data. The strategy adopts an alternating optimization framework to progressively impute missing features by iteratively minimizing an objective function. The core innovation lies in the dynamic collaboration between feature completion and graph structure optimization. This mechanism significantly improves the accuracy of node representation and attribute imputation. Extensive experiments on five different datasets demonstrate that the proposed method performs well in node classification tasks. These results confirm the effectiveness of our method in addressing missing attributes in graph data and highlight its potential value in broader graph analysis applications.

The current EGS framework is designed for homogeneous graph structures. Its alternating optimization process relies on matrix inversion, which leads to high computational complexity and poses a bottleneck for large-scale graph data. Experiment results also show that the robustness of the framework under high missing rates needs further improvement. Future work will focus on lightweight optimization methods to enable robust representation learning on heterogeneous graphs with high missing rates. We also aim to explore the practical implementation of this framework to support real-world deployment.

## Data Availability

The Cora, Citeseer, and Pubmed datasets analysed during the current study are available in the Planetoid repository, https://github.com/kimiyoung/planetoid/tree/master/data; the Amazon Computers dataset analysed during the current study is available in the PyTorch Geometric repository, https://github.com/XinPeng97/MATE/tree/main/data/amac; and the Amazon Photo dataset analysed during the current study is available in the PyTorch Geometric repository, https://github.com/XinPeng97/MATE/tree/main/data/amap.

## Appendix

### Proof of reversibility

Firstly, we observe that both the normalized Laplacian matrix and the unit matrix have symmetry. Since the addition operation of matrices maintains symmetry, their sum is a symmetric matrix, i.e., matrix $(\frac{1}{2\lambda _{1} } \Delta + \textrm{I})$ must also be a symmetric matrix. Next, we note that the normalized Laplacian matrix is semi-positive definite, which means that all its eigenvalues are non-negative. The unit matrix, on the other hand, has all eigenvalues of 1, which are all positive. Therefore, when we add the normalized Laplacian matrix to the unit matrix to obtain matrix $(\frac{1}{2\lambda _{1} } \Delta + \textrm{I})$, its eigenvalues will be the sum of the eigenvalues of the normalized Laplacian matrix and the non-negative constants, thus ensuring that all the eigenvalues of the matrix $(\frac{1}{2\lambda _{1} } \Delta + \textrm{I})$ are positive. Since matrix $(\frac{1}{2\lambda _{1} } \Delta + \textrm{I})$ is symmetric and all of its eigenvalues are positive, by the nature of symmetric positive definite matrices, we can conclude that matrix $(\frac{1}{2\lambda _{1} } \Delta + \textrm{I})$ is positive definite. Further, an important property of positive definite matrices is invertibility, i.e., the determinant of a positive definite matrix is not zero, and thus an inverse matrix exists.

In summary, we prove that the matrix $(\frac{1}{2\lambda _{1}} \Delta + \textrm{I})$ is invertible. This conclusion is derived from the matrix’s symmetry, its positive definiteness, and the invertibility of positive definite matrices.

### Symmetric preservation proof via explicit iteration

Let the initial Laplacian matrix $\Delta _0 \in \mathbb {R}^{n \times n}$ be symmetric, i.e. $\Delta _0 = \Delta _0^\top$. For the feature matrix $\textrm{X} \in \mathbb {R}^{n \times d}$, by the transpose property of matrix multiplication and the double transpose property, it follows from Eq. (11) that $\mathrm {XX^\top }$ is symmetric for any $\textrm{X}$.

11 $$\begin{aligned} \mathrm {(XX}^\top )^\top = (\textrm{X}^\top )^\top \textrm{X}^\top = \textrm{XX}^\top \end{aligned}$$

Given a scalar $\lambda _2 > 0$, applying the linearity of transposition to the subtraction operation and substituting the above result, we derive Eq. (12), which proves that the updated $\Delta$ in Eq.(10) is symmetric.

12 $$\begin{aligned} \Delta ^\top = \left( \Delta _0 - \frac{1}{4\lambda _2} \textrm{XX}^\top \right) ^\top = \Delta _0^\top - \frac{1}{4\lambda _2} (\textrm{XX}^\top )^\top = \Delta _0 - \frac{1}{4\lambda _2} \textrm{XX}^\top = \Delta \end{aligned}$$

This formally proves the symmetry preservation of the updated $\Delta$.

### Necessary and sufficient conditions for positive semi-definiteness

To ensure the positive semi-definiteness of updated $\Delta$, its minimum eigenvalue $\lambda _{\min }(\Delta )$ must satisfy $\lambda _{\min }(\Delta ) \ge 0$. We apply Weyl’s inequality to the result of the update in Eq.(10), leading to Eq. (13):

13 $$\begin{aligned} \lambda _{\min }(\Delta ) \ge \lambda _{\min }(\Delta _0) - \frac{1}{4\lambda _2} \lambda _{\max }(\textrm{XX}^\top ) \end{aligned}$$

where $\lambda _{\min }(\cdot )$ and $\lambda _{\max }(\cdot )$ denote the smallest and largest eigenvalues respectively. The positive semi-definiteness is preserved when:

14 $$\begin{aligned} \lambda _{\min }(\Delta _0) \ge \frac{1}{4\lambda _2} \lambda _{\max }(\textrm{XX}^\top ) \end{aligned}$$

This condition is experimentally achievable through proper parameter configuration.
